# Weak Interactions and Instability Cascades

**DOI:** 10.1038/srep12652

**Published:** 2015-07-29

**Authors:** Taku Kadoya, Kevin S. McCann

**Affiliations:** 1 Center for Environmental Biology and Ecosystem Studies, National Institute for Environmental Studies; 2 Department of Integrative Biology, University of Guelph.

## Abstract

Food web theory states that a weak interactor which is positioned in the food web such that it tends to deflect, or mute, energy away from a potentially oscillating consumer-resource interaction often enhances community persistence and stability. Here we examine how adding other weak interactions (predation/harvesting) on the stabilizing weak interactor alters the stability of food web using a set of well-established food web models/modules. We show that such “weak on weak” interaction chains drive an indirect dynamic cascade that can rapidly ignite a distant consumer-resource oscillator. Nonetheless, we also show that the “weak on weak” interactions are still more stable than the food web without them, and so weak interactions still generally act to stabilize food webs. Rather, these results are best interpreted to say that the degree of the stabilizing effect of a given important weak interaction can be severely compromised by other weak interactions (including weak harvesting).

Food web theory states that weak to intermediate-strength trophic links can be important in promoting community persistence and stability^1^,^2^. Here, weak links often enhance stability by muting energy flow to a potentially oscillating consumer-resource interaction. While the success of modular food web theory has motivated a desire to unite low-dimensional theory with high dimensional whole food web approaches^1^,^3^,^4^,^5^, surprisingly little work has investigated intermediate complexity modules. Here, we extend simple modular theory to higher dimensional modules by looking at the influence of multiple weak interactors on a few common focal food web modules (Fig. 1).

Although numerous mechanisms can stabilize a food web^6^, much of the modular theory relies on the fact that underlying consumer-resource interactions oscillate when consumption rates, and/or numerical responses of consumers, are large relative to consumer mortality rates^7^. Thus, any biological mechanism that acts to reduce the overall consumption rates, or numerical responses of the consumer, relative to consumer mortality acts to stabilize that interaction. Weak interactions (sensu McCann *et al.*^2^) as a stabilizing property often work by placing a weak interactor in such a position in the food web that it tends to deflect, or mute, energy away from the potential oscillator (e.g., exploitative competition; Fig. 1c). Similarly, a weak interaction weak interaction can stabilize by acting to increase the mortality on a potentially oscillating consumer-resource interaction (e.g., a 3-species food chain module; Fig. 1a). In both cases, the flux of the potentially oscillating consumer-resource sub-system is muted relative to the consumer mortality and so the system is stabilized. Here, we will refer to this stabilizing weak interactor as the stabilizing agent (Fig. 1). We note that such oscillator-stabilizing agent combinations are embedded in all of the ubiquitous food web modules to date that explore the role of weak interactions (Fig. 1).

It is interesting to note that weak interactions frequently have a strong and precipitous influence on stability. As an example, a module without the weak interaction can be enormously oscillatory while with the weak interaction the dynamics can settle into a well-bounded equilibrium dynamic. While an interesting and powerful stabilization result, this result suggests that this stabilizing weak interaction is potentially precarious—a slight alteration of the interaction may cause a significant effect on the stability of the food web. To address this, we briefly consider how adding other weak interactions may alter the dynamics and stability of low-dimensional modules.

In what follows, using a set of well-established food web models/modules, we examine how predation on the stabilizing agent alters the stability of the food web (Figs 2, 3). Similarly, we also simultaneously examine the effects of harvesting on stabilizing agents—a research area that has not yet been considered to our knowledge. We show that such “weak on weak” interactions (including weak harvesting) have an inordinate ability to destabilize a food web (sensu Keystone species; we will return to this in the discussion). Intriguingly, these weak on weak interaction chains drive an indirect dynamic cascade that can rapidly ignite a distant consumer-resource oscillator. Nonetheless, we also show that the “weak on weak” interactions are still more stable than the food web without them, which suggests that weak interactions still generally act to stabilize food webs. We end by discussing future work required in light of our finding.

## Results

For the food chain module, we find that adding a top predator (TP) that acts directly on the stabilizing agent (P) immediately destabilizes the system (Fig. 2b). We compared the autocorrelation function of the C-R oscillation in isolation (Fig. 2a) with the oscillation created by TP upon entry (Fig. 2b) and found that both have approximately the same cycle length (cycle length = 18.4; Supplementary Fig. S1) suggesting that the presence of TP reduces P and leaves the distant C-R oscillator to re-express itself. Notice that this happens immediately upon the entry of TP so that it is a weak interaction that rapidly acts to destabilize the system. This effect is made even more rapid and dramatic with harvesting (Fig. 2c). Our first result, then, is that a weak-on-weak interaction can act rapidly and powerfully to impede the stabilizing potential of a pivotal weak interaction. This effect may be particularly sensitive to harvesting suggesting that there may be multispecies cases where harvesting even modest amounts on a relatively uncommon species has the alarming effect of massively impacting the stability of a whole system.

Notice in Fig. 2b,c that we have given the C-R oscillating maxima and minima in the absence of the stabilizing agent, predator P as dashed horizontal lines (i.e., this is the underlying potential of the C-R oscillator). In both Fig. 2b (novel predator) and Fig. 2c (harvesting), the presence of the weak-on-weak interaction remains stabilizing, or equivalent, relative to the C-R oscillator alone suggesting that weak interactions, even when combined in this way, tend to remain stabilizing relative to the case where we have no weak interactions. Our second result, then, is that the overall influence of weak interactions remains stabilizing; however, the strength of this stabilization depends dramatically on the precise network structure of weak interactions.

**Figure 1:**
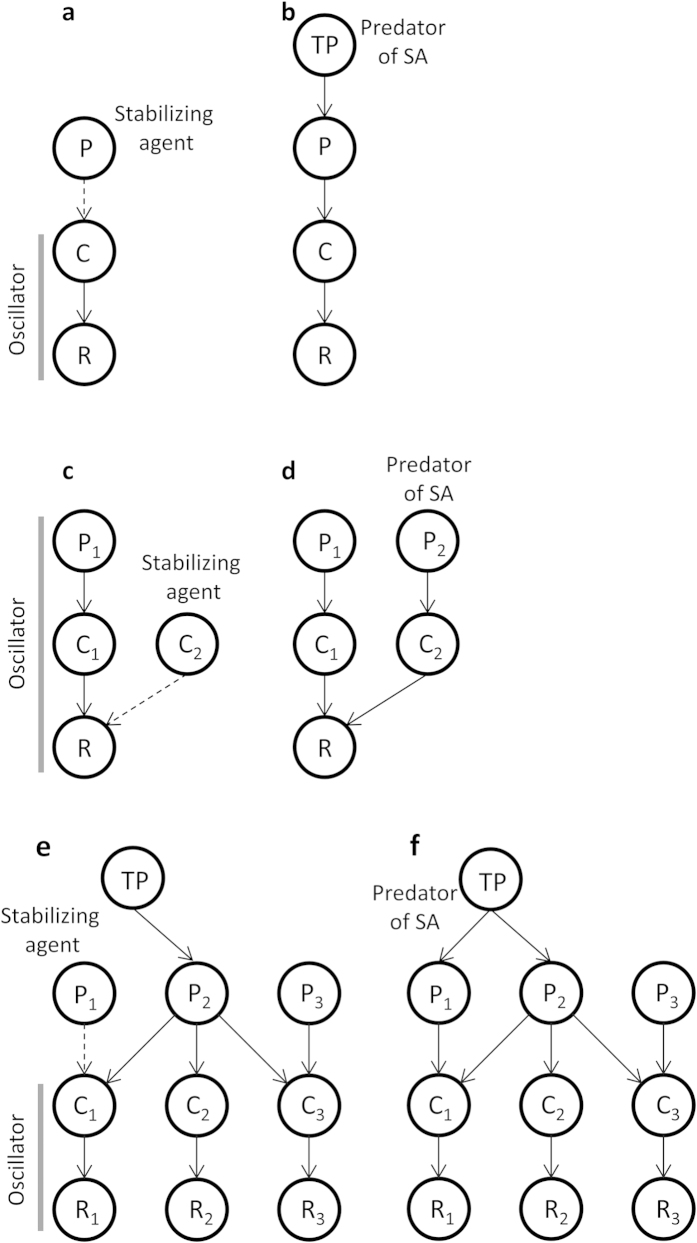
The food-web configurations. (**a**) a 3-species food chain module, (**b**) a 3-species food chain module with predation on stabilizing agent, (**c**) a food web with multiple intermediate consumers, a exploitive competition module, (**d**) a food web with multiple intermediate consumers with predation on stabilizing agent, (**e**) a complex food web consisting of 10 species, in which C_1_-R_1_ is potential oscillator, and (**f**) a complex 10-species food web with predation on stabilizing agent. In *a*, *c* and *e*, stabilizing agent is a stabilizing weak interactor in such a position in the food web that it tends to deflect, or mute, energy away from potentially oscillating strong consumer-resource interactions, denoted as oscillator. Dashed arrows represent weak interactions.

**Figure 2:**
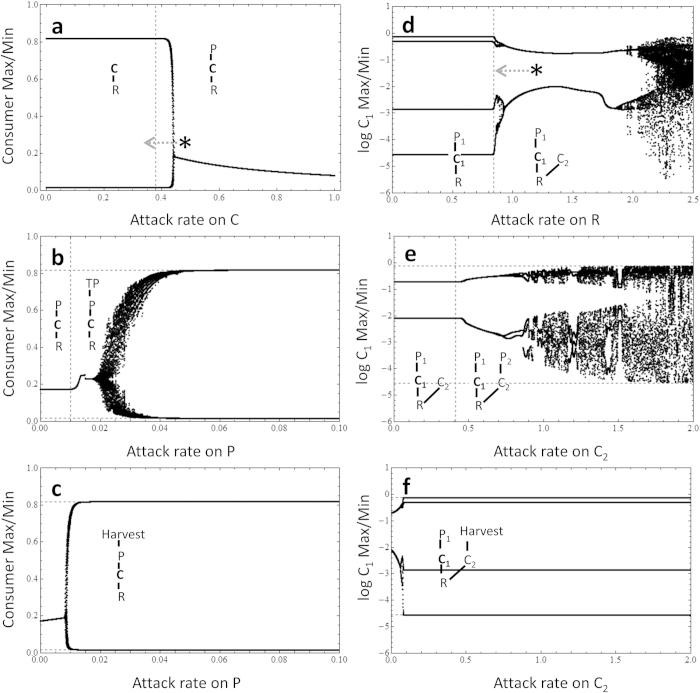
The local minima and maxima for consumer density C in a 3-species food chain module case (**a–c**) and consumer density C_1_ in a exploitive competition module case (**d–f**). (**a**) a 3-species food chain module, (**b**) one with predation on stabilizing agent P, (**c**) one with harvest on stabilizing agent, P, (**d**) a food web with multiple intermediate consumers, (**e**) one with predation on stabilizing agent, C_2_ and (**f**) one with harvest on stabilizing agent, C_2_. The 3-species food chain module case (**a–c)**: in *a*, vertical dashed line represents the attack rate (i.e., *a*_*P*_ ≈ 0.38) where P can start to invade the system. Asterisk represents attack rate of P on C used in *b* and *c*. Dashed arrow represents the direction where the state of the P-C-R module moves when top predator, TP or harvesting is added to the system as shown in *b* and *c*. In *b*, vertical dashed line represents the attack rate (i.e., *a*_*T*_ ≈ 0.01) where TP can start to invade the system. Horizontal dashed line in b and c represents the C-R oscillating maxima and minima in the absence of the top predator P. The exploitive competition module case (**d–f)**: In *d*, vertical dashed line represents the attack rate (i.e., *a*_*C2*_ ≈ 0.84) where C_2_ can start to invade the system. Asterisk represents attack rate of C_2_ on R used in *e* and *f*. Dashed arrow represents the direction where the state of the P_1_-C_1_-R-C_2_ module moves when predator, P_2_ or harvesting is added to the system as shown in *e* and *f*. In *e,* vertical line represents the attack rate (*a*_*P2*_ ≈ 0.41) where P_2_ can start to invade the system. Horizontal dashed lines in *e* and *f* represent the P_1_-C_1_-R oscillating maxima and minima in the absence of the consumer C_2_. See Supplementary Methods for parameter values.

We next extend this result to a slightly more complicated food web model with two underlying oscillators. Again, however, we first identify the potential oscillators (here, P_1_-C_1_ and C_1_-R are oscillators) and the stabilizing agent (C_2_) (Figs 1c and 2d). In this case, the stabilizing agent reduces the dynamics to a more bounded limit cycle with weak to intermediate attack rates (see dashed lines depicting local maxima and local minima for P_1_-C_1_–R attractor relative to the solid curves depicting full system local and maxima from 0.0 to 0.41 in the attack rate in Fig. 2e). As soon as the predator, P_2_, of the stabilizing agent invades the system, the food web rapidly and dramatically destabilizes. Similarly, the harvest of the stabilizing agent drives a precipitous destabilization (in this case, it causes aggressive period doubling ending in broader limit cycle with properties, not surprisingly, similar to the isolated P_1_-C_1_-R attractor). Again, the weak-on-weak interaction has a powerful destabilizing influence that cascades from the P_2_-C_2_ interaction all the way to the P_1_-C_1_ interaction (recall Fig. 1d). Although the overall effect of weak interactions remains stabilizing relative to the case of no weak interactions (Fig. 2e,f).

**Figure 3:**
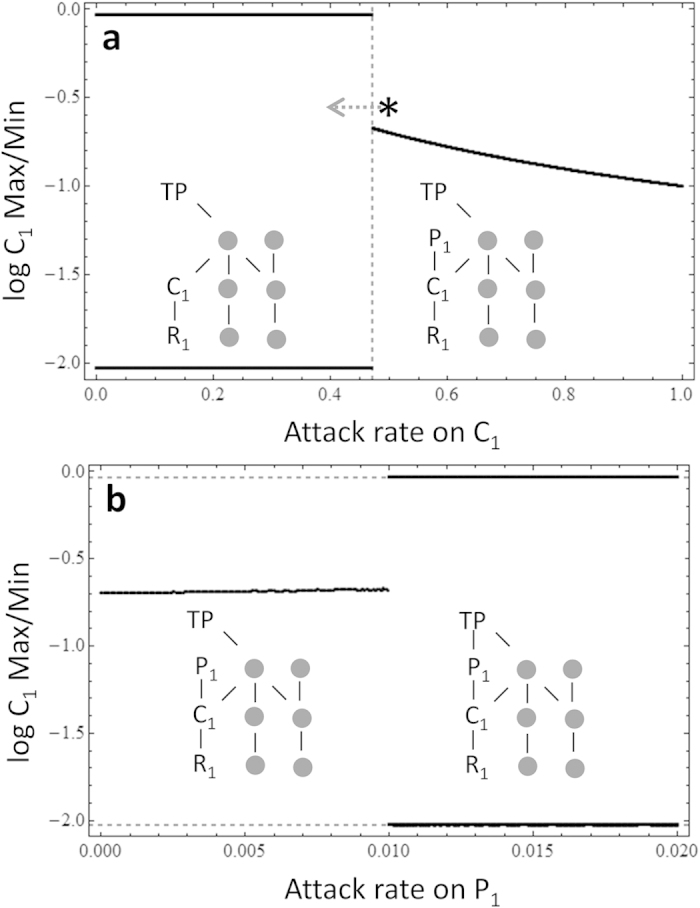
The local minima and maxima for consumer density, C_1_ in (**a**) a complex food web with/without stabilizing agent P_1_ and (**b**) that with predation on stabilizing agent by top predator, TP. In *a*, vertical dashed line represents the attack rate (i.e., *a*_*P1*_ ≈ 0.47) where P_1_ can start to invade the system. Asterisk represents attack rate of P_1_ on C_1_ which is used in *b*. Dashed arrow represents the direction where the state of the food web moves when P_1_-TP interaction is added to the system as shown in *b*. In *b,* horizontal dashed lines represent the C_1_-R_1_ oscillating maxima and minima in the absence of the predator P_1_. See Fig 1 and Supplementary Methods for variable names and parameter values.

Finally, to show that this result can manifest just as readily in high diversity webs we performed a numerical experiment on a complex food web case. In this food web, the potential oscillator, C_1_-R_1,_ is first stabilized by a weak interaction from P_1_ (Fig. 3a). We then added a link from top predator, TP, to P_1_, and again we found a precipitous destabilization of the system for a weakly interacting TP-P_1_ interaction (Fig. 3b). We compared the autocorrelation function of the C_1_-R_1_ oscillation in isolation (before P_1_ enters in Fig. 3a) with the oscillation that arises with the addition of the TP-P_1_ interaction (Fig. 3b) and found that both cycles had approximately the same cycle length (cycle length = 24.1; Supplementary Fig. S2), suggesting that the weak interactor, TP reduces P_1_ and leaves the distant C_1_-R_1_ oscillator to re-express itself. Our third result, then, is that weak-on-weak interactions can ignite a distant instability cascade even in complex food webs.

## Discussion

Here, we have shown that “weak-on-weak interactions” can quickly destabilize the system by negating the influence of a stabilizing agent in a simple food web module. The mechanism lying behind the phenomena is simple: the interactor (weak or strong) on the stabilizing agent impedes its ability to shunt away energy from the potential oscillator, thus allowing that oscillator to express itself.

Nonetheless, it is important to point out that the combined effect of the weak-on-weak interactions still yield a stabilizing effect compared to the unstable sub-system module without them. The theory, therefore, remains consistent with previous results on weak interactions, but points out that the extent of weak interaction muting can be seriously altered by the arrangement of weak interactions in a diverse system. We found the same results repeatedly in other common motifs^8^, a complex food web with predation at lower trophic level as well as models with more realistic parameters based on metabolic allometry (Supplementary Figs S3-S7). We also found that harvesting had more immediate, and debilitating, destabilizing effects on food web modules than predation. This effect suggests that even extremely weak harvesting on a rare species (that happens to be a stabilizing agent) can potentially have a large effect on system stability.

Our results were done on relatively simple food web models, as well as shown to work at different trophic levels in much more complicated higher diversity models (Supplementary Figs S3-S7), suggesting that the destabilizing potential of a weak-on-weak interaction may exist in complex natural food webs. It remains to ask how this result is influenced by realistic food web structure. Recent work using whole matrix theory suggests that the stabilizing effect of weak interaction ought to remain in whole webs^9^. If this is true, it is possible that such “weak-on-weak” instability cascades documented here are muted, on average, by a realistic diverse set of interactions, or these long chains of effects are buffered in complex webs^10^. Note, the network motifs examined here, such as the 3-species chains (Fig. 1a) and the diamond food web (Fig. S3) are known to be over-represented in natural food webs^8^,^11^. It may be essential, based on our study, to examine how often those motifs contain oscillator interactions (and stabilizing agents as well) within them in order to predict how the natural food webs respond to the addition of new interactions in terms of stability. Unfortunately, while we have a solid understanding of food web topology^12^ and some of the major flows in aggregated pathways^13^,^14^,^15^, we have little understanding of the intricate structure of interactions in whole networks. Nonetheless, it remains of interest to ask if such potent destabilization remains in more complex networks, especially if weak harvesting on a rare species can drive a destabilizing cascade.

Research on keystone species suggests that such potent stabilizing agents exist^16^. The removal of any one species, in these cases, has an inordinate impact on community dynamics and diversity^17^. Similarly, the theory discussed here suggests that a weak novel interaction (or harvesting) could impede these same keystone species in a manner that causes rapid cascading instability. Also, ample research has found that top predators, or large-bodied higher trophic level species, often have disproportional stability consequences in complex food webs^18^,^19^. If so, these species may also be candidates for strong harvesting-driven instability cascades. Further, invasions have been responsible for the continuous addition of novel interactions. Our results suggest that even a small population of invasive species can cause cascading instabilities in a food web if the species establishes predatory interaction with a stabilizing agent in an indigenous food web. The results here, on simple models, are suggestive of a keystone food web theory in simple modules. Future research needs to examine whether the instability cascades mediated by weak interactions, as found in the present study, are dampened or amplified in a realistically complex food web. Identifying such key interactions is paramount when these systems are under threat of multiple human impacts.

## Methods

### The food web models

All models are derived from the well-known Rosenzweig-MacArthur food chain equations^20^ (also see Fig. 1 and Supplementary Methods). The simple 3-species food chains (Fig. 1a) is formulated as follows:


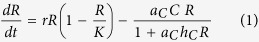



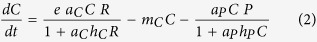



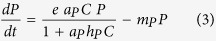


where, R, is the resource density, C, is the consumer density, P, is the predator density, *r* is the intrinsic growth rate of the resource, K is the carrying capacity, *a*_*i*_ is the attack rate of species *i*, *h*_*i*_ is the handling time of species *i*, *m*_*i*_ is the mortality rate of species *i* and *e* is the assimilation rate. Similarly, we generated a food web with multiple intermediate consumers (exploitive competition module; Fig. 1c) and a complex food web which consists of 3 resources, each of them are consumed by 3 different consumers that are predated by 3 predators, and one of the predators is predated by a top predator (Fig. 1e). The full description of the model is shown in Supplementary Methods.

### Numerical experiments

Here, we show our result for three food web cases, a food chain (Fig. 1a,b) and exploitative competition (Fig. 1c,d) modules and a complex food web (Fig. 1e,f), although the results generally extend to arbitrarily large models. In all numerical experiments we start from a food web parameterized in a manner similar to ref. 2 in that we have a weak interaction muting a potentially strong and oscillatory interaction. In Fig. 1a, then, the P-C interaction mutes a potentially strong C-R interaction leaving the P-C-R in a stable equilibrium. Similarly, in Fig. 1c, the R-C_2_ interaction mutes potentially strong C_1_-R and P_1_-C_1_ interactions leaving the module in a bounded limit cycle. Also, in Fig. 1e, P_1_-C_1_ interaction mutes a potentially strong C_1_-R_1_ interaction leaving the P_1_-C_1_-R_1_ in a stable equilibrium.

This parameterization allows us to easily: (i) identify the “potential oscillator” (e.g., C-R), and; (ii) identify the interaction (e.g., P-C) that is muting the potential oscillator and so stabilizing the system (referred to as the stabilizing agent). We then introduce a novel interaction (e.g., TP-P) that acts directly on the stabilizing agent and ask how changes in interaction strength (via attack rate) of this novel interaction influence system stability. The precise procedure for the experiment is as follows:(1) Set parameters so that food web becomes stable under combination with potential oscillator and stabilizing agent (Fig. 1a,c,e), and; (2) Add a predator (or harvesting term) on the stabilizing agent (Fig. 1b,d,f), and investigate the non-equilibrium stability (local maxima and minima) as a function of the interaction strength (attack rate) or harvesting rate of the novel interaction. Note, the harvesting interaction has no dynamics (Supplementary Methods).

## Additional Information

**How to cite this article**: Kadoya, T. and McCann, K. S. Weak Interactions and Instability Cascades. *Sci. Rep.* **5**, 12652; doi: 10.1038/srep12652 (2015).

## Supplementary Material

Supplementary Information
