# Supplementary material for: Weak Interactions and Instability Cascades
Source: Sci Rep. 2015 Jul 29;5:12652. doi: 10.1038/srep12652 (PMC4518242; doi:10.1038/srep12652)
Supplement: Supplementary Information [file srep12652-s1.pdf]

# Weak Interactions and Instability Cascades

Taku Kadoya & Kevin S. McCann

## Supplementary Information

## 1. Supplementary Figures

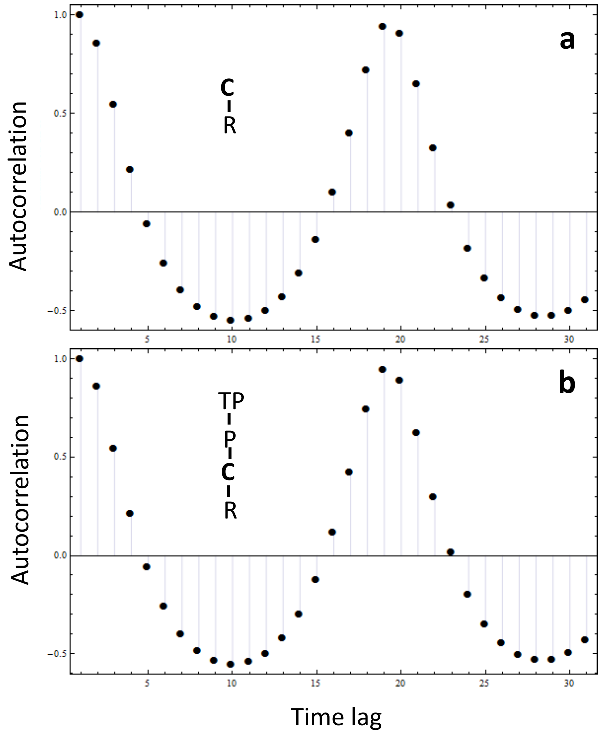


Figure S1. Autocorrelation function of consumer in (a) C-R relationships and (b) a 3-species food chain with top predator, TP on stabilizing agent, P. Cycle length is ca. 18.4 both in (a) and (b). Parameter values correspond to those in Figs. 2a, b.

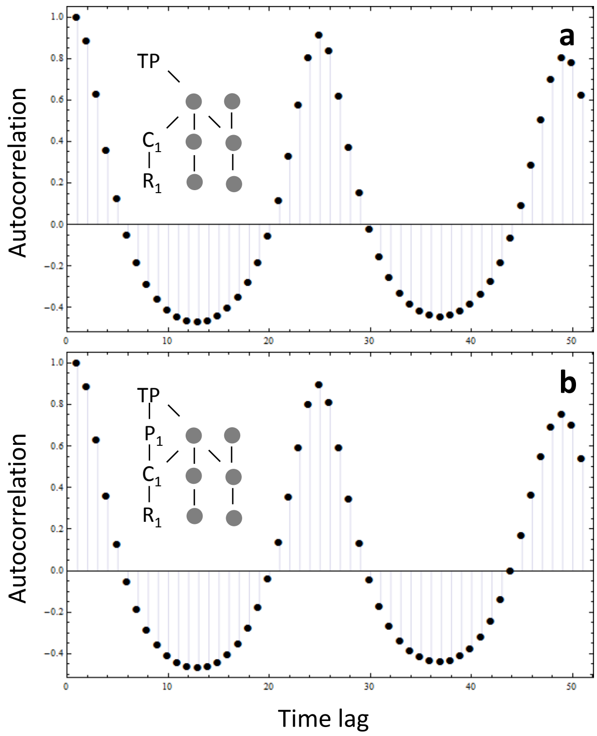


Figure S2. Autocorrelation function of consumer, $C_1$ in (a) a 9-species food web and (b) one with stabilizing agent, P1 and its predator, TP (10-specie food web). Cycle length is ca. 24.1 both in (a) and (b). Parameter values correspond to those in Fig. 3.

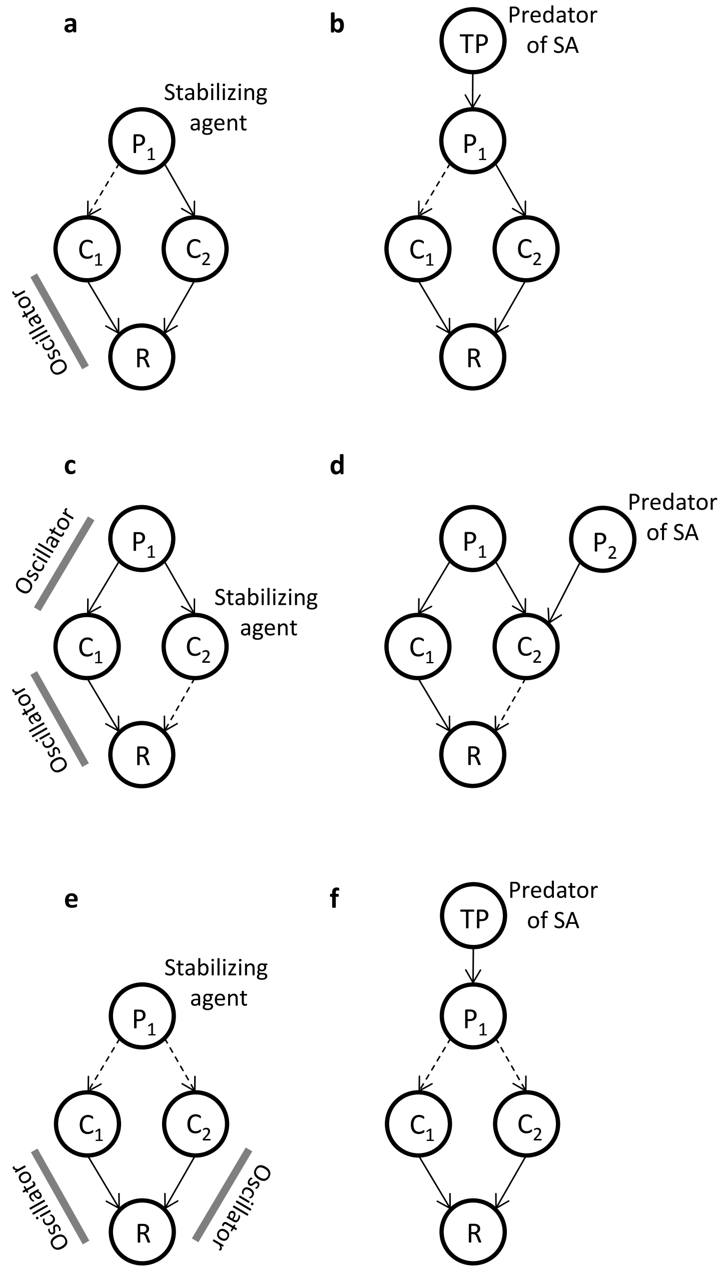


Figure S3. The diamond food-web configurations. In *a*, *c* and *e*, stabilizing agent is a stabilizing weak interactor in such a position in the food web that it tends to deflect, or mute, energy away from potentially oscillating strong consumer-resource interactions, denoted as oscillator. Dashed arrows represent weak interactions. In *b, d* and *f*, a predator on the stabilizing agent is added to the food web *a*, *c* and *e*, respectively.

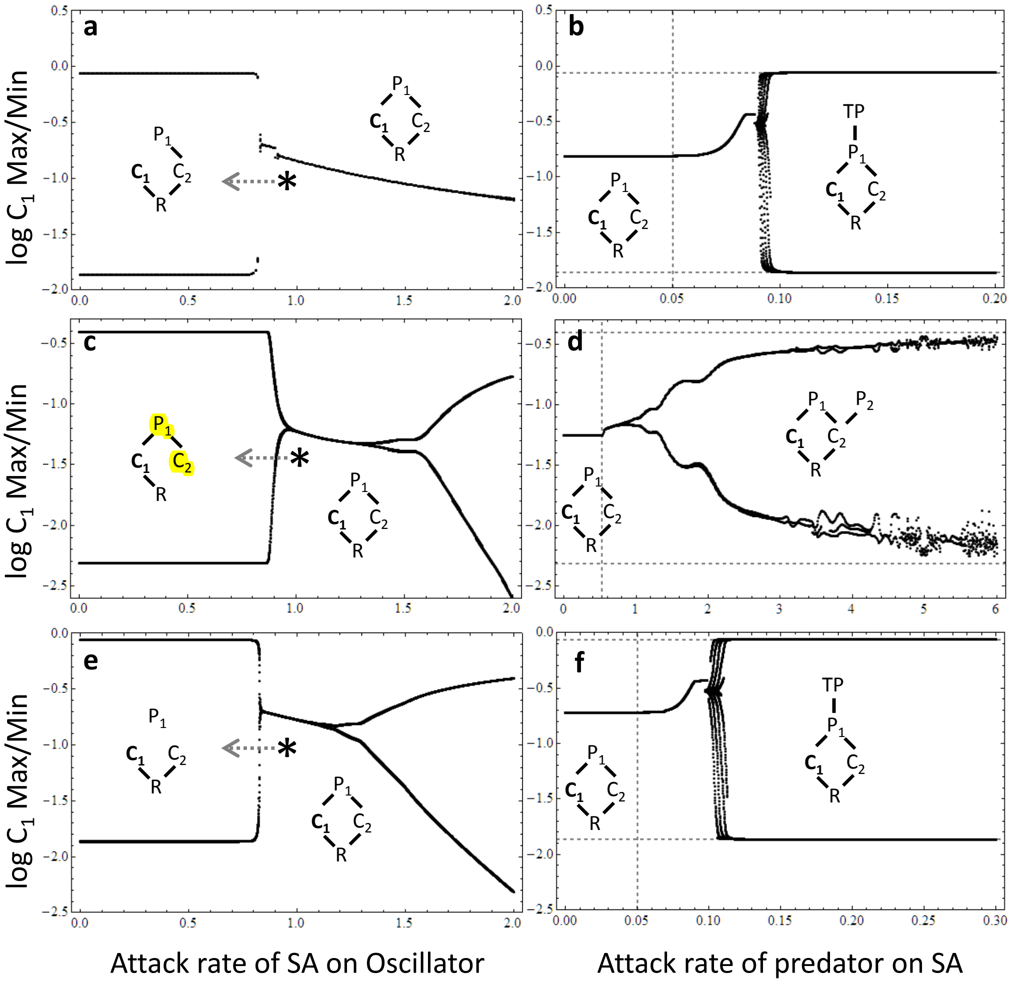


Figure S4. The local minima and maxima for consumer density $C_1$ in the diamond food webs with different position of oscillator interaction(s) (see Fig. S3). In *a*, *c* and *e*, immediately after stabilizing agent invades the system by increasing its attack rate, the system becomes stable. In *b*, *d* and *f*, attack rate of stabilizing agent shown as asterisk in *a*, *c* and *e* is used, respectively, and attack rate of the predator on stabilizing agent is increased gradually. Immediately after the predator starts to invade, the system becomes unstable. Vertical lines represent the attack rate where TP or $P_2$ can start to invade the system. Horizontal dashed lines in *b, d* and *f* represent the oscillating maxima and minima in the absence of stabilizing agent in *a*, *c* and *e*, respectively. See Supplementary Methods for parameter values.

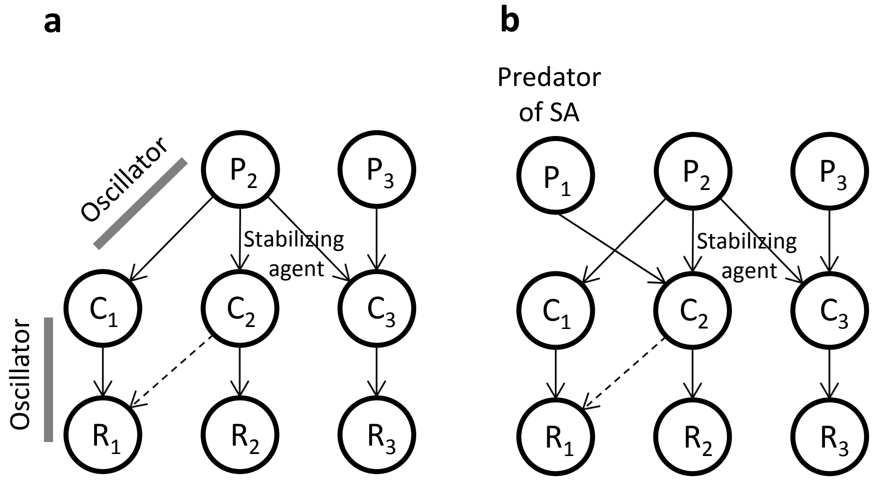


Figure S5. The 8 species food web configurations. In *a*, stabilizing agent, $C_2$ is a stabilizing weak interactor in such a position in the food web that it tends to deflect, or mute, energy away from potentially oscillating strong consumer-resource interactions, denoted as oscillator. Dashed arrows represent weak interactions. In *b*, a predator, $P_1$ on the stabilizing agent is added to the food web.

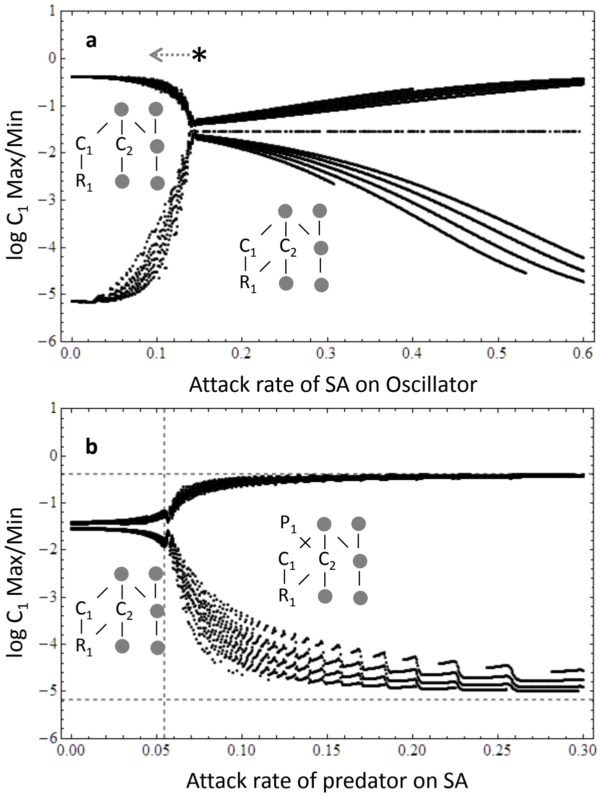


Figure S6. The local minima and maxima for consumer density $C_1$ in the complex food web with 8 species (see Fig. S5). In *a*, immediately after stabilizing agent, $C_2$ establishes the interaction with $R_1$ by increasing its attack rate, the system becomes relatively stable. In *b*, the attack rate of stabilizing agent, $C_2$ shown as asterisk in *a*, is used and attack rate of predator, $P_1$ on the stabilizing agent is gradually increased. Immediately after the predator starts to invade, the system becomes unstable. Vertical line represents the attack rate where $P_1$ can start to invade the system. Horizontal dashed lines in *b* represent the oscillating maxima and minima in the absence of stabilizing agent in *a*. See Supplementary Methods for parameter values.

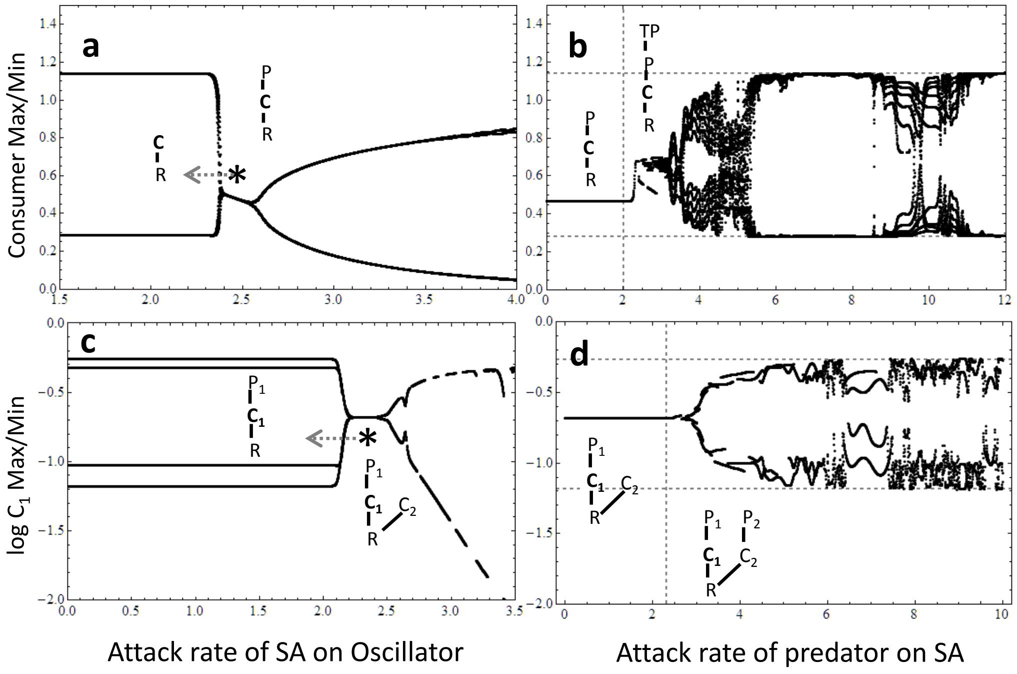


Figure S7. The local minima and maxima for consumer density C in a 3-species food chain module case (a, b) and consumer density $C_1$ in a exploitive competition module case (c, d) with model parameters based on metabolic allometry. (a) a 3-species food chain module, (b) one with predation on stabilizing agent P, (c) a food web with multiple intermediate consumers and (d) one with predation on stabilizing agent, $C_2$. **The 3-species food chain module case (a, b)**: in *a*, asterisk represents attack rate of P on C used in *b*. Dashed arrow represents the direction where the state of the P-C-R module moves when top predator, TP is added to the system as shown in *b*. In *b*, vertical dashed line represents the attack rate (i.e., $y_T \approx 2.0$) where TP can start to invade the system. Horizontal dashed lines in b represent the C-R oscillating maxima and minima in the absence of the top predator P. **The exploitive competition module case (c, d)**: In *c*, asterisk represents attack rate of $C_2$ on R used in *d*. Dashed arrow represents the direction where the state of the $P_1$-$C_1$-R-$C_2$ module moves when predator, $P_2$ or harvesting is added to the system as shown in *d*. In *d*, vertical line represents the attack

rate ($y_{P2} \approx 2.3$) where $P_2$ can start to invade the system. Horizontal dashed lines in *d* represent the $P_1$-$C_1$-R oscillating maxima and minima in the absence of the consumer $C_2$. See Supplementary Methods for model structures and parameter values.

## 2. Supplementary Methods

### 2.1 The food-web models analyzed in the main text

All models are derived from the well-known Rosenzweig-MacArthur food chain equations[20], where R, is the resource density, C, is the consumer density, P, is the predator density, $r$ is the intrinsic growth rate of the resource, K is the carrying capacity, $a_i$ is the attack rate of species $i$, $h_i$ is the handling time of species $i$, $m_i$ is the mortality rate of species $i$ and $e$ is the assimilation rate. The models are specified as follows:

***a1. A 3-species food chain***

$$\frac{dR}{dt} = rR\left(1 - \frac{R}{K}\right) - \frac{a_C C\ R}{1 + a_C h_C R}$$

$$\frac{dC}{dt} = \frac{e\ a_C C\ R}{1 + a_C h_c R} - m_C C - \frac{a_P C\ P}{1 + a_P h_P C}$$

$$\frac{dP}{dt} = \frac{e\ a_P C\ P}{1 + a_P h_P C} - m_P P$$

Parameter values used in Fig. 2a are: $r$ = 1.0, K = 1.0, $e$ = 1.0, $a_C$ = 5.0, $h_C$ = 0.5, $h_P$ = 0.1, $m_C$ = 0.4 and $m_P$ = 0.08. In Fig. 2a, $a_P$ was changed gradually.

***a2. A 3-species food chain with predation on stabilizing agent***

$$\frac{dR}{dt} = r\ R\left(1 - \frac{R}{K}\right) - \frac{a_C C\ R}{1 + a_C h_C R}$$

$$\frac{dC}{dt} = \frac{e\ a_C C\ R}{1 + a_C h_C R} - m_C C - \frac{a_P C\ P}{1 + a_P h_P C}$$

$$\frac{dP}{dt} = \frac{e\ a_P C\ P}{1 + a_P h_P C} - m_P P - \frac{a_T P\ T}{1 + a_T h_T P}$$

$$\frac{dT}{dt} = \frac{e\, a_T P\, T}{1 + a_T h_T P} - m_T T$$

Parameter values used in Fig. 2b are: $r$ = 1.0, K = 1.0, $e$ = 1.0, $a_C$ = 5.0, $a_P$ = 0.47, $h_C$ = 0.5, $h_P$ = 0.1, $h_T$ = 0.05, $m_C$ = 0.4, $m_P$ = 0.08 and $m_T$ = 0.01. In Fig. 2b, $a_T$ was changed gradually.

***a3. A 3-species food chain with harvest on stabilizing agent***

$$\frac{dR}{dt} = r\, R\left(1 - \frac{R}{K}\right) - \frac{a_C C\, R}{1 + a_C h_C R}$$

$$\frac{dC}{dt} = \frac{e\, a_C C\, R}{1 + a_C h_C R} - m_C C - \frac{a_P C\, P}{1 + a_P h_P C}$$

$$\frac{dP}{dt} = \frac{e\, a_P C\, P}{1 + a_P h_P C} - m_P P - \frac{a_H P}{1 + a_H h_H P}$$

Parameter values used in Fig. 2c are: $r$ = 1.0, K = 1.0, $e$ = 1.0, $a_C$ = 5.0, $a_P$ = 0.47, $h_C$ = 0.5, $h_P$ = 0.1, $h_H$ = 0.05, $m_C$ = 0.4 and $m_P$ = 0.08. In Fig. 2c, $a_H$ was changed gradually.

***b1. A food web with multiple intermediate consumers***

$$\frac{dR}{dt} = r\, R\left(1 - \frac{R}{K}\right) - \frac{a_{C_1} C_1\, R}{1 + a_{C_1} h_{C_1} R} - \frac{a_{C_2} C_2\, R}{1 + a_{C_2} h_{C_2} R}$$

$$\frac{dC_1}{dt} = \frac{e\, a_{C_1} C_1\, R}{1 + a_{C_1} h_{C_1} R} - m_{C_1} C_1 - \frac{a_{P_1} C_1\, P_1}{1 + a_{P_1} h_{P_1} C_1}$$

$$\frac{dC_2}{dt} = \frac{e\, a_{C_2} C_2\, R}{1 + a_{C_2} h_{C_2} R} - m_{C_2} C_2$$

$$\frac{dP_1}{dt} = \frac{e\, a_{P_1} C_1\, P_1}{1 + a_{P_1} h_{P_1} C_1} - m_{P_1} P_1$$

Parameter values used in Fig. 2d are: $r$ = 1.0, K = 2.0, $e$ = 0.5, $a_{C1}$ = 5.0, $a_{P1}$ = 4.0, $h_{C1}$ = 0.5, $h_{C2}$ = 0.5, $h_{P1}$ = 0.5, $m_{C1}$ = 0.4, $m_{C2}$ = 0.4 and $m_{P1}$ = 0.1. In Fig. 2d, $a_{C2}$ was changed

gradually.

***b2. A food web with multiple intermediate consumers with predation on stabilizing agent***

$$\frac{dR}{dt} = r\,R\left(1-\frac{R}{K}\right) - \frac{a_{C_1}C_1\,R}{1+a_{C_1}h_{C_1}R} - \frac{a_{C_2}C_2\,R}{1+a_{C_2}h_{C_2}R}$$

$$\frac{dC_1}{dt} = \frac{e\,a_{C_1}C_1\,R}{1+a_{C_1}h_{C_1}R} - m_{C_1}C_1 - \frac{a_{P_1}C_1\,P_1}{1+a_{P_1}h_{P_1}C_1}$$

$$\frac{dC_2}{dt} = \frac{e\,a_{C_2}C_2\,R}{1+a_{C_2}h_{C_2}R} - m_{C_2}C_2 - \frac{a_{P_2}C_2\,P_2}{1+a_{P_2}h_{P_2}C_2}$$

$$\frac{dP_1}{dt} = \frac{e\,a_{P_1}C_1\,P_1}{1+a_{P_1}h_{P_1}C_1} - m_{P_1}P_1$$

$$\frac{dP_2}{dt} = \frac{e\,a_{P_2}C_2\,P_2}{1+a_{P_2}h_{P_2}C_2} - m_{P_2}P_2$$

Parameter values used in Fig. 2e are: $r$ = 1.0, K = 2.0, $e$ = 0.5, $a_{C1}$ = 5.0, $a_{C2}$ = 1.2, $a_{P1}$ = 4.0, $h_{C1}$ = 0.5, $h_{C2}$ = 0.5, $h_{P1}$ = 0.5, $h_{P2}$ = 0.1, $m_{C1}$ = 0.4, $m_{C2}$ = 0.4, $m_{P1}$ = 0.1 and $m_{P2}$ = 0.1. In Fig. 2e, $a_{P2}$ was changed gradually.

***b3. A food web with multiple intermediate consumers with harvest on stabilizing agent***

$$\frac{dR}{dt} = r\,R\left(1-\frac{R}{K}\right) - \frac{a_{C_1}C_1\,R}{1+a_{C_1}h_{C_1}R} - \frac{a_{C_2}C_2\,R}{1+a_{C_2}h_{C_2}R}$$

$$\frac{dC_1}{dt} = \frac{e\,a_{C_1}C_1\,R}{1+a_{C_1}h_{C_1}R} - m_{C_1}C_1 - \frac{a_{P_1}C_1\,P_1}{1+a_{P_1}h_{P_1}C_1}$$

$$\frac{dC_2}{dt} = \frac{e\,a_{C_2}C_2\,R}{1+a_{C_2}h_{C_2}R} - m_{C_2}C_2 - \frac{a_H C_2}{1+a_H h_H C_2}$$

$$\frac{dP_1}{dt} = \frac{e\, a_{P_1} C_1\, P_1}{1 + a_{P_1} h_{P_1} C_1} - m_{P_1} P_1$$

Parameter values used in Fig. 2f are: $r$ = 1.0, K = 2.0, $e$ = 0.5, $a_{C1}$ = 5.0, $a_{C2}$ = 1.2, $a_{P1}$ = 4.0, $h_{C1}$ = 0.5, $h_{C2}$ = 0.5, $h_{P1}$ = 0.5, $h_H$ = 0.1, $m_{C1}$ = 0.4, $m_{C2}$ = 0.4 and $m_{P1}$ = 0.1. In Fig. 2f, $a_H$ was changed gradually.

***c. A complex food web with 10 species***

$$\frac{dR_1}{dt} = R_1(1 - R_1) - \frac{a_{C_1} C_1\, R_1}{1 + a_{C_1} h_{C_1} R_1}$$

$$\frac{dR_2}{dt} = R_2(1 - R_2) - \frac{a_{C_2} C_2\, R_2}{1 + a_{C_2} h_{C_2} R_2}$$

$$\frac{dR_3}{dt} = R_3(1 - R_3) - \frac{a_{C_3} C_3\, R_3}{1 + a_{C_3} h_{C_3} R_3}$$

$$\frac{dC_1}{dt} = \frac{a_{C_1} C_1\, R_1}{1 + a_{C_1} h_{C_1} R_1} - m_{C_1} C_1 - \frac{a_{P_1}\, P_1 C_1}{1 + a_{P_1} h_{P_1} C_1} - \frac{a_{P_{21}} C_1\, P_2}{1 + a_{P_{21}} h_{P_{21}} C_1 + a_{P_{22}} h_{P_{22}} C_2 + a_{P_{23}} h_{P_{23}} C_3}$$

$$\frac{dC_2}{dt} = \frac{a_{C_2} C_2\, R_2}{1 + a_{C_2} h_{C_2} R_2} - m_{C_2} C_2 - \frac{a_{P_{22}} C_2\, P_2}{1 + a_{P_{21}} h_{P_{21}} C_1 + a_{P_{22}} h_{P_{22}} C_2 + a_{P_{23}} h_{P_{23}} C_3}$$

$$\frac{dC_3}{dt} = \frac{a_{C_3} C_3\, R_3}{1 + a_{C_3} h_{C_3} R_3} - m_{C_3} C_3 - \frac{a_{P_{23}} C_3\, P_2}{1 + a_{P_{21}} h_{P_{21}} C_1 + a_{P_{22}} h_{P_{22}} C_2 + a_{P_{23}} h_{P_{23}} C_3} - \frac{a_{P_3} C_3\, P_3}{1 + a_{P_3} h_{P_3} C_3}$$

$$\frac{dP_1}{dt} = \frac{a_{P_1}\, P_1 C_1}{1 + a_{P_1} h_{P_1} C_1} - m_{P_1} P_1 - \frac{a_{T_1} P_1 T}{1 + a_{T_1} h_{T_1} P_1 + a_{T_2} h_{T_2} P_2}$$

$$\frac{dP_2}{dt} = \frac{a_{P_{21}} C_1 P_2 + a_{P_{22}} C_2 P_2 + a_{P_{23}} C_3 P_2}{1 + a_{P_{21}} h_{P_{21}} C_1 + a_{P_{22}} h_{P_{22}} C_2 + a_{P_{23}} h_{P_{23}} C_3} - m_{P_2} P_2 - \frac{a_{T_2} P_2 T}{1 + a_{T_1} h_{T_1} P_1 + a_{T_2} h_{T_2} P_2}$$

$$\frac{dP_3}{dt} = \frac{a_{P_3} C_3\ P_3}{1 + a_{P_3} h_{P_3} C_3} - m_{P_3} P_3$$

$$\frac{dT}{dt} = \frac{a_{T_1} P_1 T + a_{T_2} P_2 T}{1 + a_{T_1} h_{T_1} P_1 + a_{T_2} h_{T_2} P_2} - m_T T$$

Parameter values used in Fig. 3a (without $P_1$-TP interaction) are: $a_{C1}$ = 5.0, $a_{C2}$ = 4.0, $a_{C3}$ = 4.0, $a_{P21}$ = 0.1, $a_{P22}$ = 0.7, $a_{P23}$ = 0.1, $a_{P3}$ = 0.5, $a_{T2}$ = 0.15, $h_{C1}$ = 0.5, $h_{C2}$ = 0.1, $h_{C3}$ = 0.1, $h_{P1}$ = 0.05, $h_{P21}$ = 0.05, $h_{P22}$ = 0.05, $h_{P23}$ = 0.05, $h_{P3}$ = 0.05, $h_{T2}$ = 0.01, $m_{C1}$ = 0.28, $m_{C2}$ = 0.28, $m_{C3}$ = 0.28, $m_{P1}$ = 0.1, $m_{P2}$ = 0.1, $m_{P3}$ = 0.07 and $m_T$ = 0.02. In Fig. 3a, $a_{P1}$ was changed gradually. Parameter values used in Fig. 3b (with $P_1$-TP interaction) are: $a_{C1}$ = 5.0, $a_{C2}$ = 4.0, $a_{C3}$ = 4.0, $a_{P1}$ = 0.5, $a_{P21}$ = 0.1, $a_{P22}$ = 0.7, $a_{P23}$ = 0.1, $a_{P3}$ = 0.5, $a_{T2}$ = 0.15, $h_{C1}$ = 0.5, $h_{C2}$ = 0.1, $h_{C3}$ = 0.1, $h_{P1}$ = 0.05, $h_{P21}$ = 0.05, $h_{P22}$ = 0.05, $h_{P23}$ = 0.05, $h_{P3}$ = 0.05, $h_{T1}$ = 0.01, $h_{T2}$ = 0.01, $m_{C1}$ = 0.28, $m_{C2}$ = 0.28, $m_{C3}$ = 0.28, $m_{P1}$ = 0.1, $m_{P2}$ = 0.1, $m_{P3}$ = 0.07 and $m_T$ = 0.02. In Fig. 3b, $a_{T1}$ was changed gradually.

## 2.2 The models for diamond food webs

We conducted the experiment using the diamond food web modules with the same procedure as those shown in the text (Figs. 1, 2). We considered three cases where position of oscillator(s) is different: (1) a diamond food-web with consumer-resource oscillator (Fig. S3a), (2) one with cascading oscillators (Fig. S3c) and (3) one with competing oscillators (Fig. S3e). The models are specified below.

### *a. A diamond food-web*

$$\frac{dR}{dt} = r\ R\left(1 - \frac{R}{K}\right) - \frac{a_{C_1} C_1\ R}{1 + a_{C_1} h_{C_1} R} - \frac{a_{C_2} C_2\ R}{1 + a_{C_2} h_{C_2} R}$$

$$\frac{dC_1}{dt} = \frac{e\, a_{C_1} C_1\, R}{1 + a_{C_1} h_{C_1} R} - m_{C_1} C_1 - \frac{a_{P_{11}} C_1\, P_1}{1 + a_{P_{11}} h_{P_{11}} C_1 + a_{P_{12}} h_{P_{12}} C_2}$$

$$\frac{dC_2}{dt} = \frac{e\, a_{C_2} C_2\, R}{1 + a_{C_2} h_{C_2} R} - m_{C_2} C_2 - \frac{a_{P_{12}} C_2\, P_1}{1 + a_{P_{11}} h_{P_{11}} C_1 + a_{P_{12}} h_{P_{12}} C_2}$$

$$\frac{dP_1}{dt} = \frac{e\, (a_{P_{11}} C_1\, P_1 + a_{P_{12}} C_2\, P_1)}{1 + a_{P_{11}} h_{P_{11}} C_1 + a_{P_{12}} h_{P_{12}} C_2} - m_{P_1} P_1$$

(1) In the diamond food-web with consumer-resource oscillator case (Fig. S4a), R-$C_1$ is the oscillator. Parameter values used are: $r$ = 1.0, K = 2.0, $e$ = 0.5, $a_{C1}$ = 5.0, $a_{C2}$ = 1.26, $a_{P12}$ = 0.08, $h_{C1}$ = 0.5, $h_{C2}$ = 0.5, $h_{P11}$ = 0.3, $h_{P\,12}$= 0.3, $m_{C1}$ = 0.4, $m_{C2}$ = 0.4 and $m_{P1}$ = 0.08. In Fig. S4a, $a_{P11}$ was changed gradually. (2) In the diamond food-web with cascading oscillators (Fig. S4c), R-$C_1$-$P_1$ are the oscillators. Parameter values used are: $r$ = 1.0, K = 2.0, $e$ = 0.5, $a_{C1}$ = 5.0, $a_{P11}$ = 2.0, $a_{P12}$ = 0.15, $h_{C1}$ = 0.5, $h_{C2}$ = 0.5, $h_{P11}$ = 0.3, $h_{P\,12}$= 0.3, $m_{C1}$ = 0.4, $m_{C2}$ = 0.4 and $m_{P1}$ = 0.08. In Fig. S4c, $a_{C2}$ was changed gradually. (3) In the diamond food web with competing oscillators (Fig. S4e), R-$C_1$ and R-$C_2$ are the oscillators. Parameter values used are: $r$ = 1.0, K = 2.0, $e$ = 0.5, $a_{C1}$ = 5.0, $a_{C2}$ = 4.92, $h_{C1}$ = 0.5, $h_{C2}$ = 0.5, $h_{P11}$ = 0.3, $h_{P\,12}$= 0.3, $m_{C1}$ = 0.4, $m_{C2}$ = 0.4 and $m_{P1}$ = 0.08. In Fig. S4e, Both $a_{P11}$ and $a_{P12}$ were changed gradually.

***b. A diamond food web with consumer-resource oscillator with predation on stabilizing agent***

$$\frac{dR}{dt} = r\, R\left(1 - \frac{R}{K}\right) - \frac{a_{C_1} C_1\, R}{1 + a_{C_1} h_{C_1} R} - \frac{a_{C_2} C_2\, R}{1 + a_{C_2} h_{C_2} R}$$

$$\frac{dC_1}{dt} = \frac{e\, a_{C_1} C_1\, R}{1 + a_{C_1} h_{C_1} R} - m_{C_1} C_1 - \frac{a_{P_{11}} C_1\, P_1}{1 + a_{P_{11}} h_{P_{11}} C_1 + a_{P_{12}} h_{P_{12}} C_2}$$

$$\frac{dC_2}{dt} = \frac{e\, a_{C_2} C_2\, R}{1 + a_{C_2} h_{C_2} R} - m_{C_2} C_2 - \frac{a_{P_{12}} C_2\, P_1}{1 + a_{P_{11}} h_{P_{11}} C_1 + a_{P_{12}} h_{P_{12}} C_2}$$

$$\frac{dP_1}{dt} = \frac{e\ (a_{P_{11}} C_1\ P_1 + a_{P_{12}} C_2\ P_1)}{1 + a_{P_{11}} h_{P_{11}} C_1 + a_{P_{12}} h_{P_{12}} C_2} - m_{P_1} P_1 - \frac{a_T T P_1}{1 + a_T h_T P_1}$$

$$\frac{dT}{dt} = \frac{e a_T T P_1}{1 + a_T h_T P_1} - m_T T$$

Parameter values used in Fig. S4b are: $r$ = 1.0, K = 2.0, $e$ = 0.5, $a_{C1}$ = 5.0, $a_{C2}$ = 1.26, $a_{P11}$ =*0.94,* $a_{P12}$ = 0.08, $h_{C1}$ = 0.5, $h_{C2}$ = 0.5, $h_{P11}$ = 0.3, $h_{P12}$= 0.3, $m_{C1}$ = 0.4, $m_{C2}$ = 0.4 and $m_{P1}$ = 0.08. In Fig. S4b, $a_T$ was changed gradually.

***c. A diamond food web with cascading oscillators with predation on stabilizing agent***

$$\frac{dR}{dt} = r\ R\left(1 - \frac{R}{K}\right) - \frac{a_{C_1} C_1\ R}{1 + a_{C_1} h_{C_1} R} - \frac{a_{C_2} C_2\ R}{1 + a_{C_2} h_{C_2} R}$$

$$\frac{dC_1}{dt} = \frac{e\ a_{C_1} C_1\ R}{1 + a_{C_1} h_{C_1} R} - m_{C_1} C_1 - \frac{a_{P_{11}} C_1\ P_1}{1 + a_{P_{11}} h_{P_{11}} C_1 + a_{P_{12}} h_{P_{12}} C_2}$$

$$\frac{dC_2}{dt} = \frac{e\ a_{C_2} C_2\ R}{1 + a_{C_2} h_{C_2} R} - m_{C_2} C_2 - \frac{a_{P_{12}} C_2\ P_1}{1 + a_{P_{11}} h_{P_{11}} C_1 + a_{P_{12}} h_{P_{12}} C_2} - \frac{a_{P_2} C_2 P_2}{1 + a_{P_2} h_{P_2} P_2}$$

$$\frac{dP_1}{dt} = \frac{e\ (a_{P_{11}} C_1\ P_1 + a_{P_{12}} C_2\ P_1)}{1 + a_{P_{11}} h_{P_{11}} C_1 + a_{P_{12}} h_{P_{12}} C_2} - m_{P_1} P_1$$

$$\frac{dP_2}{dt} = \frac{e\ a_{P_2} C_2 P_2}{1 + a_{P_2} h_{P_2} P_2} - m_{P_2} P_2$$

Parameter values in Fig. S4d used are: $r$ = 1.0, K = 2.0, $e$ = 0.5, $a_{C1}$ = 5.0, $a_{C2}$ = 1.05, $a_{P11}$ = 2.0, $a_{P12}$ = 0.15, $h_{C1}$ = 0.5, $h_{C2}$ = 0.5, $h_{P11}$ = 0.3, $h_{P12}$= 0.3, $m_{C1}$ = 0.4, $m_{C2}$ = 0.4 and $m_{P1}$ = 0.08. In Fig. S4d, $a_{P2}$ was changed gradually.

***d. A diamond food web with competing oscillators with predation on stabilizing agent***

$$\frac{dR}{dt} = r\ R\left(1 - \frac{R}{K}\right) - \frac{a_{C_1} C_1\ R}{1 + a_{C_1} h_{C_1} R} - \frac{a_{C_2} C_2\ R}{1 + a_{C_2} h_{C_2} R}$$

$$\frac{dC_1}{dt} = \frac{e\,a_{C_1} C_1\,R}{1 + a_{C_1} h_{C_1} R} - m_{C_1} C_1 - \frac{a_{P_{11}} C_1\,P_1}{1 + a_{P_{11}} h_{P_{11}} C_1 + a_{P_{12}} h_{P_{12}} C_2}$$

$$\frac{dC_2}{dt} = \frac{e\,a_{C_2} C_2\,R}{1 + a_{C_2} h_{C_2} R} - m_{C_2} C_2 - \frac{a_{P_{12}} C_2\,P_1}{1 + a_{P_{11}} h_{P_{11}} C_1 + a_{P_{12}} h_{P_{12}} C_2}$$

$$\frac{dP_1}{dt} = \frac{e\,(a_{P_{11}} C_1\,P_1 + a_{P_{12}} C_2\,P_1)}{1 + a_{P_{11}} h_{P_{11}} C_1 + a_{P_{12}} h_{P_{12}} C_2} - m_{P_1} P_1 - \frac{a_T T P_1}{1 + a_T h_T P_1}$$

$$\frac{dT}{dt} = \frac{e a_T T P_1}{1 + a_T h_T P_1} - m_T T$$

Parameter values used in Fig. S4f are: $r$ = 1.0, K = 2.0, $e$ = 0.5, $a_{C1}$ = 5.0, $a_{C2}$ = 4.92, $a_{P11}$ = 0.88, $a_{P12}$ = 0.88, $h_{C1}$ = 0.5, $h_{C2}$ = 0.5, $h_{P11}$ = 0.3, $h_{P\,12}$= 0.3, $m_{C1}$ = 0.4, $m_{C2}$ = 0.4 and $m_{P1}$ = 0.08. In Fig. S4f, $a_T$ was changed gradually.

### 2.3 The model for a complex food web with 8 species

We conducted the experiment using a complex food web model with 8 species (Fig. S5a) with the same procedure as those shown in the text (Figs. 1, 3). The model is specified as follows:

***a. A complex food web with 8 species with predation on stabilizing agent***

$$\frac{dR_1}{dt} = R_1(1 - R_1) - \frac{a_{C_1} C_1\,R_1}{1 + a_{C_1} h_{C_1} R_1} - \frac{a_{C_{21}} C_{21}\,R_1}{1 + a_{C_{21}} h_{C_{21}} R_1 + a_{C_{22}} h_{C_{22}} R_2}$$

$$\frac{dR_2}{dt} = R_2(1 - R_2) - \frac{a_{C_{22}} C_{22}\,R_2}{1 + a_{C_{21}} h_{C_{21}} R_1 + a_{C_{22}} h_{C_{22}} R_2}$$

$$\frac{dR_3}{dt} = R_3(1 - R_3) - \frac{a_{C_3} C_3\,R_3}{1 + a_{C_3} h_{C_3} R_3}$$

$$\frac{dC_1}{dt} = \frac{a_{C_1} C_1\,R_1}{1 + a_{C_1} h_{C_1} R_1} - m_{C_1} C_1 - \frac{a_{P_{21}} C_1\,P_2}{1 + a_{P_{21}} h_{P_{21}} C_1 + a_{P_{22}} h_{P_{22}} C_2 + a_{P_{23}} h_{P_{23}} C_3}$$

$$\frac{dC_2}{dt} = \frac{e(a_{C_{21}} C_{21}\, R_1 + a_{C_{22}} C_{22}\, R_2)}{1 + a_{C_{21}} h_{C_{21}} R_1 + a_{C_{22}} h_{C_{22}} R_2} - m_{C_2} C_2$$

$$- \frac{a_{P_{22}} C_2\, P_2}{1 + a_{P_{21}} h_{P_{21}} C_1 + a_{P_{22}} h_{P_{22}} C_2 + a_{P_{23}} h_{P_{23}} C_3}$$

$$\frac{dC_3}{dt} = \frac{a_{C_3} C_3\, R_3}{1 + a_{C_3} h_{C_3} R_3} - m_{C_3} C_3 - \frac{a_{P_{23}} C_3\, P_2}{1 + a_{P_{21}} h_{P_{21}} C_1 + a_{P_{22}} h_{P_{22}} C_2 + a_{P_{23}} h_{P_{23}} C_3}$$

$$- \frac{a_{P_3} C_3\, P_3}{1 + a_{P_3} h_{P_3} C_3}$$

$$\frac{dP_1}{dt} = \frac{a_{P_1}\, P_1 C_2}{1 + a_{P_1} h_{P_1} C_2} - m_{P_1} P_1$$

$$\frac{dP_2}{dt} = \frac{e(a_{P_{21}} C_1 P_2 + a_{P_{22}} C_2 P_2 + a_{P_{23}} C_3 P_2)}{1 + a_{P_{21}} h_{P_{21}} C_1 + a_{P_{22}} h_{P_{22}} C_2 + a_{P_{23}} h_{P_{23}} C_3} - m_{P_2} P_2$$

$$\frac{dP_3}{dt} = \frac{a_{P_3} C_3\, P_3}{1 + a_{P_3} h_{P_3} C_3} - m_{P_3} P_3$$

Parameter values used in Fig. S6a (i.e., without $C_2$-$P_1$ interaction) are: $a_{C1}$ = 5.0, $a_{C22}$ = 0.3, $a_{C3}$ = 0.5, $a_{P21}$ = 3.0, $a_{P22}$ = 0.01, $a_{P23}$ = 0.01, $a_{P3}$ = 0.43, $h_{C1}$ = 0.5, $h_{C21}$ = 0.5, $h_{C22}$ = 0.1, $h_{C3}$ = 0.1, $h_{P21}$ = 0.05, $h_{P22}$ = 0.05, $h_{P23}$ = 0.05, $h_{P3}$ = 0.05, $m_{C1}$ = 0.28, $m_{C2}$ = 0.28, $m_{C3}$ = 0.28, $m_{P2}$ = 0.1 and $m_{P3}$ = 0.07. In Fig. S6a, $a_{C21}$ was changed gradually.

Parameter values used in Figs S6b (i.e., with $C_2$-$P_1$ interaction) are: $a_{C1}$ = 5.0, $a_{C21}$ = 0.14, $a_{C22}$ = 0.3, $a_{C3}$ = 0.5, $a_{P21}$ = 3.0, $a_{P22}$ = 0.01, $a_{P23}$ = 0.01, $a_{P3}$ = 0.43, $h_{C1}$ = 0.5, $h_{C21}$ = 0.5, $h_{C22}$ = 0.1, $h_{C3}$ = 0.1, $h_{P1}$ = 0.05, $h_{P21}$ = 0.05, $h_{P22}$ = 0.05, $h_{P23}$ = 0.05, $h_{P3}$ = 0.05, $m_{C1}$ = 0.28, $m_{C2}$ = 0.28, $m_{C3}$ = 0.28, $m_{P1}$ = 0.07, $m_{P2}$ = 0.1 and $m_{P3}$ = 0.07. In Fig. S6b, $a_{P1}$ was changed gradually.

## 2.4 The food web models with parameters based on metabolic allometry

We conducted the same experiment with those shown in Figs 1a,b,c,d and Figs. 2a,b,d,e based on the models formulated using the allometric parameterization of McCann and Yodzis[21] as follows:

***a1. A 3-species food chain***

$$\frac{dR}{dt} = R(1 - R) - \frac{x_C y_C C\ R}{R + R_0}$$

$$\frac{dC}{dt} = \frac{x_C y_C C\ R}{R + R_0} - x_C C - \frac{x_P y_P C\ P}{C + C_0}$$

$$\frac{dP}{dt} = \frac{x_P y_P C\ P}{C + C_0} - x_P P$$

where, R, is the resource density, C, is the consumer density, P, is the predator density, $x_i$ is the mass-specific metabolic rate of species *i*, $y_i$ is a measure of ingestion rate per unit metabolic rate of species *i*, and $R_0$ and $C_0$ are the half saturation density for the consumer and predator functional responses, respectively. Note that in this model, carrying capacity is standardized (i.e., equals one) and thus omitted. Parameter values used in Fig. S7a are: $R_0 = 0.5$, $C_0 = 0.7$, $x_c = 0.2$, $y_c = 4.2$ and $x_p = 0.15$. In Fig. S7a, $y_p$ was changed gradually.

***a2. A 3-species food chain with predation on stabilizing agent***

$$\frac{dR}{dt} = R(1 - R) - \frac{x_C y_C C\ R}{R + R_0}$$

$$\frac{dC}{dt} = \frac{x_C y_C C\ R}{R + R_0} - x_C C - \frac{x_P y_P C\ P}{C + C_0}$$

$$\frac{dP}{dt} = \frac{x_P y_P C\ P}{C + C_0} - x_P P - \frac{x_T y_T P\ T}{P + P_0}$$

$$\frac{dT}{dt} = \frac{x_T y_T P\ T}{P + P_0} - x_T T$$

Parameter values used in Fig. S7b are: $R_0 = 0.5$, $C_0 = 0.7$, $P_0 = 0.7$, $x_c = 0.2$, $y_c = 4.2$, $x_p = 0.15$, $y_p = 2.5$ and $x_t = 0.09$. In Fig. S7b, $y_t$ was changed gradually.

***b1. A food web with multiple intermediate consumers***

$$\frac{dR}{dt} = R(1-R) - \frac{x_{C_1} y_{C_1} C_1\ R}{R + R_{1\_0}} - \frac{x_{C_2} y_{C_2} C_2\ R}{R + R_{2\_0}}$$

$$\frac{dC_1}{dt} = \frac{x_{C_1} y_{C_1} C_1\ R}{R + R_{1\_0}} - x_{C_1} C_1 - \frac{x_{P_1} y_{P_1} C_1\ P_1}{C_1 + C_{1\_0}}$$

$$\frac{dC_2}{dt} = \frac{x_{C_2} y_{C_2} C_2\ R}{R + R_{2\_0}} - x_{C_2} C_2$$

$$\frac{dP_1}{dt} = \frac{x_{P_1} y_{P_1} C_1\ P_1}{C_1 + C_{1\_0}} - x_{P_1} P_1$$

Parameter values used in Fig. S7c are: $x_{c1} = 0.4$, $y_{c1} = 2.18$, $x_{p1} = 0.08$, $y_{p1} = 3.4$, $x_{c2} = 0.2$, $R_{1\_0} = 0.3$, $R_{2\_0} = 0.8$ and $C_{1\_0} = 0.5$. In Fig. S7c, $y_{c2}$ was changed gradually.

***b2. A food web with multiple intermediate consumers with predation on stabilizing agent***

$$\frac{dR}{dt} = R(1-R) - \frac{x_{C_1} y_{C_1} C_1\ R}{R + R_{1\_0}} - \frac{x_{C_2} y_{C_2} C_2\ R}{R + R_{2\_0}}$$

$$\frac{dC_1}{dt} = \frac{x_{C_1} y_{C_1} C_1\ R}{R + R_{1\_0}} - x_{C_1} C_1 - \frac{x_{P_1} y_{P_1} C_1\ P_1}{C_1 + C_{1\_0}}$$

$$\frac{dC_2}{dt} = \frac{x_{C_2} y_{C_2} C_2\ R}{R + R_{2\_0}} - x_{C_2} C_2 - \frac{x_{P_2} y_{P_2} C_2\ P_2}{C_2 + C_{2\_0}}$$

$$\frac{dP_1}{dt} = \frac{x_{P_1} y_{P_1} C_1\ P_1}{C_1 + C_{1\_0}} - x_{P_1} P_1$$

$$\frac{dP_2}{dt} = \frac{x_{P_2} y_{P_2} C_2\ P_2}{C_2 + C_{2\_0}} - x_{P_2} P_2$$

Parameter values used in Fig. S7d are: $x_{c1}$ = 0.4, $y_{c1}$ = 2.18, $x_{p1}$ = 0.08, $y_{p1}$ = 3.4, $x_{c2}$ = 0.2, $y_{c2}$ = 2.3, $R_{1\_0}$ = 0.3, $R_{2\_0}$ = 0.8, $C_{1\_0}$ = 0.5, $C_{2\_0}$ = 0.5 and $x_{p2}$ = 0.08. In Fig. S7d, $y_{p2}$ was changed gradually.

## References


21. McCann, K. & Yodzis, P. Biological conditions for chaos in a 3-species food-chain. *Ecology* **75**, 561-564, doi:10.2307/1939558 (1994).
